# The Association between Weight for Gestational Age and Kidney Volume: A Study in Newborns in India

**Published:** 2013-01-29

**Authors:** Kirtisudha Mishra, Vikram Datta, Aarushi Aarushi, Maninder Kaur Narula, R. Subramanyam Iyer, Sushma Nangia

**Affiliations:** 1 Department of Pediatrics; 2 Department of Neonatology, Lady Hardinge Medical College, New Delhi, India; 3 Department of Radiology, Kalawati Saran Children's Hospital

**Keywords:** Small for Gestational Age, Newborn, Kidney, Low Birth Weight

## Abstract

***Objective:*** The association of low birth weight (LBW) with adult onset diseases like hypertension is suggested to be partially mediated by a low number of nephrons at birth. Studies have established a relation between LBW and renal volume as the latter is a surrogate marker of total nephron number. Most such studies have considered birth weight or gestational age as separate independent predictors, without taking into consideration the baby’s weight with respect to its gestational age. This study aims to investigate the influence of weight for gestational age on kidney volume in newborns.

***Methods:*** Consecutive newborns delivered in the department of neonatology in a tertiary care medical college and hospital, were included in a cross-sectional study. The subjects were classified as appropriate for gestational age (AGA) and small for gestational age (SGA) as per Lubchenco’s charts of weight for gestational age (WGA). Bilateral kidney dimensions were measured by a single observer and combined kidney volumes were calculated and compared between the groups.

***Findings*** ***:*** Four hundred and seventeen newborns (SGA 159; AGA 258) were included. The mean combined kidney volume (CKV) was significantly lower among SGA newborns (13.85±4.02 cm^3^) compared to that of AGA (16.88±4.53 cm^3^) (*P*<.001). Univariable and multivariable analyses were done for assessing the effect of demographic, anthropometric and maternal parameters on CKV. WGA, crown heel length, gestational age and postnatal age (hours of life) were independent predictors of mean CKV. An SGA newborn was expected to have a mean CKV 1.57 cm less (95% CI -2.49 cm to -0.65 cm) than that of its AGA counterpart.

***Conclusion:*** Considering the future implications of being SGA and having low kidney volumes at birth, it is essential to have an objective depiction of the relationship between these two vital parameters. This study from the Indian subcontinent brings forth such an association.

## Introduction

Low birth weight (LBW) has been identified as a risk factor for development of adult-onset diseases, including hypertension, type 2 diabetes and poor renal course. Barker et al and Brenner et al hypothesized a deficit in nephron number to be the explanation for this fetal programming of adult disease^[^^1^^-^^4^^]^. Several animal models, epidemiological and retrospective human studies have demonstrated that the association of LBW with subsequent hypertension is mediated, at least in part, by a congenital deficit in nephron number^[^^5^^-^^9^^]^. The number of glomeruli correlates with renal mass, but both these parameters cannot be measured in vivo. So, renal volume, which is proportional to renal mass, has been used as a surrogate marker of low nephron number ^[^^10^^,^^11^^]^.

 It has been seen in studies that renal volume is lower in children and adults who had low birth weight compared to those who had normal birth weight^[^^10^^, ^^12^^]^. Antenatal ultrasonography of human fetuses have shown that intrauterine growth retardation is associated with reduced kidney volume^[^^13^^-^^15^^]^. However, most such studies assessing renal volume have considered birth weight or gestational age as separate independent predictors, without taking into consideration the baby’s weight with respect to its gestational age. This study aims to investigate the influence of weight for gestational age on kidney volume in newborns.

## Subjects and Methods

A cross-sectional study was conducted in the department of neonatology in a tertiary care medical college and hospital. Ethical approval was obtained from the institutional ethical committee. After taking informed consent, consecutive newborns delivered in the same hospital were screened for inclusion into the study. The exclusion criteria were major congenital anomalies, severe birth asphyxia (defined as Apgar score at 5 minutes ≤3 and/or cord pH ≤7.0), any major illness with hemodynamic instability, known acute kidney injury (defined as an abrupt (<48 hrs) reduction of kidney function manifesting as urine output <0.5ml/kg/hr for >6 hours and/or increase in serum creatinine of ≥0.3mg/dl or ≥50%), use of nephrotoxic drugs in the immediate postnatal period and refusal of consent.

 The details of the included subjects like gestational age, birth weight, gender, hours of postnatal life, Apgar score, head circumference, crown-heel length and crown-rump length were recorded. Birth weight, obtained from birth records, was taken by a digital weighing scale to the nearest 0.005 kg. Body length was measured supine with an infantometer to the nearest 0.1 cm. The gestational age was determined by the date of last menstrual period, antenatal ultrasound and confirmed by Modified Ballard’s scoring system^[^^16^^]^ The subjects were then classified as appropriate for gestational age (AGA), small for gestational age (SGA) or large for gestational age (LGA) based on Lubchenco’s reference charts for assessment of weight for gestational age^[^^17^^]^. SGA was defined as weight below the 10th percentile for gestational age, AGA as weight between10th and 90th percentile and LGA as weight above the 90th percentile for gestational age as per Lubchenco’s reference charts^[^^18^^]^. Ponderal index (PI) defined as weight in grams/length^[^^3^^]^ was calculated for those infants identified as SGA^[^^19^^]^. The SGA infants were then further classified into two groups: low PI (PI <10^th^ percentile for gestational age) and appropriate PI (PI between 10th and 90th percentile) as per Lubchenco’s Ponderal index-gestational age distribution^[^^20^^]^. Some maternal parameters like parity, antenatal hemoglobin, and known medical or surgical illness were also obtained from maternal records.

 All the included subjects underwent ultrasonological evaluation for kidney size. Ultrasound was done by a single observer who was blinded to the anthropometric parameters of the subject. Kidney size was determined by USG using 3.5- 5 MHZ sector probe. For examination of right kidney child was placed in left lateral position and vice versa for left kidney. The kidney was identified in the sagittal plane along its longitudinal axis. In this position, longitudinal measurements of the largest length were performed. The probe was then rotated 90 degrees and cross-sectional anteroposterior measurements of the width and depth at the hilar level were performed. All dimensions were measured to the nearest 0.1 cm in both kidneys. Kidney volume was then calculated in cubic centimeters using the equation of an ellipsoid: volume = mean length ×mean width × mean depth × 0.523^[^^21^^]^. 

 Descriptive statistics like mean and standard deviations for continuous variables and frequencies for categorical variables were calculated. The mean combined (right and left) kidney volume (CKV) was compared among AGA, SGA and LGA groups of infants by ANOVA test of significance with post-hoc Tukey test for individual comparisons. 

**Table 1:** Demographic and anthropometric characteristics of newborns

**Characteristics**	**Mean (SD** # **)/Frequency (n)**
**Gender: Male/Female (n)**	223/194
**Birth weight (Kg)**	2.387 (0.648)
**Gestational Age (weeks)**	37.38 (2.65)
**Term/Preterm (n)**	282/ 135
**Postnatal age (hours of life)**	39.68 (28.10)
**Weight for Age: SGA/AGA/LGA** * ** (n)**	159/239/19

# SD: Standard deviation;

* SGA: Small for gestational age, AGA: Appropriate for gestational age, LGA: Large for gestational age; n:number

Univariable and multivariable linear regression analyses were done to assess the effect of confounders. The multivariable linear regression model was made taking those parameters which were found to be significant in univariable analysis, excluding the ones showing co linearity. Model performance was judged by adjusted R^2^. The correlation between mean combined kidney volume and significant continuous variables was also determined. Significance was taken at a *P* value of 0.05. All statistical analysss was done by SPSS version 16.

## Findings

A total of 472 newborns were screened for inclusion and exclusion criteria. Six of them had congenital malformations, 36 were admitted in the immediate postnatal period due to serious illness and in 13 cases parents refused consent. Thus, 417 neonates were included, 14 twins and 403 singletons. The characteristics of the newborns are shown in Table 1. Out of 159 newborns that were SGA, 107 had a low PI and 52 had appropriate PI. The mean combined kidney volume (CKV) was significantly lower among SGA newborns (13.85±4.02 cm^3^) compared to that of AGA (16.88±4.53 cm^3^) and LGA (20.99±5.87 cm^3^) subjects (*P*<.001). Further comparison of CKV between the subgroups of SGA (low versus appropriate PI) failed to show any significant difference. As the number of LGA infants was minimal (19), further analyses were done taking LGA and AGA together as one composite group and named AGA for convenience. Some demographic, anthropometric and maternal parameters were assessed by univariable analysis for their effect on the mean CKV (Table 2). Among the variables tested, weight for gestational age, birth weight, gestational age, gender, postnatal age, crown heel and crown rump length were found to have significant association with CKV. In order to eliminate the secondary effect of different parameters acting together to influence kidney volume, we constructed multivariable regression models. 

**Table 2:** Univariable regression analysis assessing the effect of demographic, anthropometric and maternal parameters on the mean combined kidney volume of newborns

**Variables**	**Regression co-efficient**	***P*** **-value**	**95% Confidence Interval**
**Birth weight**	4.99	<0.001	4.47- 5.52
**Gestational age (GA)**	0.77	<0.001	0.61 - 0 .93
**Prematurity (GA <37 weeks)**	-3.72	<0.001	-4.63 - - 2.807
**Male gender **	1.15	0.01	0.24- 2.07
**Postnatal age (hours of life)**	-0.03	<0.001	-0.05 - -0.01
**Small for gestational age **	-3.33	<0.001	-4.22 - -2.44
**Head circumference**	1.36	<0.001	1.16 - 1.55
**Crown heel length**	0.89	<0.001	0.79 – 1.00
**Crown rump length**	0.95	<0.001	0.78 - 1.13
**Mother’s parity**	0.35	0.09	-0.05 - 0 .75
**Antenatal maternal hemoglobin**	0.31	0.1	-0.06 - 0.68

**Table 3:** Multivariable regression analysis assessing effect of demographic, anthropometric and maternal parameters on the mean combined kidney volume of newborns

**Variables**	**Regression co-efficient**	***P-*** **value**	**95% Confidence Interval**
**Small for gestational age **	-1.57	0.001	-2.49 - -0.65
**Gender**	0.60	0.09	-0.10 - 1.31
**Gestational age**	0.23	0.04	0.01 - 0.44
**Hours of postnatal life**	0.01	0.05	0.00 - .027
**Crown-heel length**	0.56	<0.001	0.39 - 0.74

Model R square=0.441

The parameters which were significant were included in this multivariable model, excluding the ones which were collinear (Table 3). It was found that weight for gestational age (SGA versus AGA), crown heel length, gestational age and postnatal age (hours of life) were independent predictors of mean CKV. An SGA newborn was expected to have a mean CKV 1.57 cm less (95% CI -2.49 cm to -0.65 cm) than that of its AGA counterpart.

## Discussion

This study establishes an objective relationship between the kidney sizes of SGA and AGA newborns. Most studies till now have deduced equations relating the kidney size with crude birth weight or gestational age separately without showing the relationship with weight for gestational age (WGA). 

 As evidence is increasing regarding the role of the kidney and the number of nephrons at birth in determining diseases in later life, it is imperative to identify markers of nephron number for risk stratification and monitoring^[^^1^^-^^9^^,^^22^^]^. Researchers have recognized low birth weight, kidney size, prematurity and short stature as some of the clinical surrogates of nephron number^[^^10^^,^^23^^,^^24^^]^. 

 Many studies in literature have shown that low birth weight is associated with reduced kidney size^[^^10^^,^^13^^,^^26^^]^. Spencer et al estimated an increase in kidney volume of about 15 ml/1.73 m^2^ for every kilogram increase in birth weight. Their study had adjusted for the effect of age and gender, but not for gestational age^10^. Similarly, birth weight was found to be an independent predictor of the glomerular number, in an autopsy study of kidneys^[^^27^^]^. However, the kidneys of older children or adults were measured and compared between those who had low birth weight and those who had normal birth weight^[^^10^^,^^26^^-^^29^^]^. The size of the kidneys in older ages may be influenced by environmental and extraneous factors and may not reflect solely the impact of birth weight^[^^30^^]^. Also, the size of the kidney in later ages may in no way be related to the number of nephrons at birth, as glomerulii may undergo adaptive changes like hypertrophy. The effect of intrauterine growth on the kidneys is best manifested at birth. Moreover, in the studies mentioned above, the gestational ages at birth were not available, so the contribution of prematurity on kidney size could not be ascertained in such studies. 

 Various observations have established a relation between kidney volume and gestational age, working out mathematical models to calculate the association. Such an association was also evident from our study, with an expected increase of 0.23 cm in CKV for every week increase in gestational age. Many of the studies showing this relationship have been done by antenatal ultrasonography of fetal kidneys, without considering the secondary effect of other factors like fetal weight, IUGR^[^^31^^, ^^32^^]^. Some authors have investigated the kidney size by antenatal ultrasound taking into account the weight for gestational age^[^^13^^-^^15^^]^. It was observed that renal volume in the intrauterine growth-restricted fetuses was less than that in the group of fetuses that were not intrauterine growth restricted after adjusting for gestational age^[^^13^^]^. Though WGA has been considered here, the fetal weight taken in these studies is either an estimated one, or, the classification into SGA and AGA has been done after birth. 

 The length/height of a child is another parameter which has been found to be a surrogate marker of renal size, though, this has mostly been studied in older children and hence the effect of IUGR or SGA could not be assessed^[^^33^^,^^34^^]^. Our study established an independent association between crown-heel length of the newborn and renal size.

 It has been shown that the renal size increases with increasing postnatal age^[^^25^^]^. This has also been observed as a rapid catch-up growth in SGA newborns up to 3 months of age, by Giapros et al^[^^35^^]^. It has been shown in our study, that irrespective of the influence of other variables, the kidney volume increases by 0.05 cm for every increasing hour of postnatal life. 

 It is essential to establish a quantitative relationship between neonatal renal volume and birth weight of the newborn with respect to his gestational age or, in other words, weight for gestational age. Such a difference in kidney size between SGA and AGA newborns has hardly been studied. Giapros et al were one of the few research groups who identified smaller kidneys in SGA infants compared with the AGA infants^[^^35^^]^. However, this study dealt with only one aspect of renal size (renal length), without taking the entire kidney volume into consideration. It was demonstrated in a study by Schmidt et al, that WGA had a stronger influence than crude birth weight or gestational age on CKV at birth^[^^25^^]^. They suggested that analyses of the impact of intrauterine growth retardation on kidney size or function should take into account WGA, instead of birth weight alone. Our study brings forth such an association. It shows that after adjusting for secondary effect of other variables (gender, gestational age, postnatal age and crown-heel length) an SGA newborn was expected to have a mean CKV 1.57 cm less (95% CI -2.49 cm to -0.65 cm) than that of its AGA counterpart.

 Studies assessing newborn kidney size and its determinants have deduced correlations between somatic parameters and different dimensions of the kidney size like length, depth or width. Many researchers have shown interest particularly in kidney length^[^^35^^-^^39^^]^. Our study has dealt with the composite combined (left and right) kidney volume, which should be a better indicator of renal mass or total nephron number, rather than any single dimension. Keeping in mind the future risk implication of total nephron number at birth, determinants of kidney volume would be more practically useful.

 In the present study there was no significant difference in kidney volumes between those with low PI or asymmetrical IUGR versus those with appropriate PI or symmetrical IUGR, indicating that all SGA newborns have low kidney volumes, irrespective of the time of intrauterine insult. 

 Since the proposal of the fact that intrauterine kidney growth is strongly associated with morbidity in later life, a lot of interest has been shown by researchers in investigating these parameters in communities where low birth weight is common^[^^10^^,^^27^^,^^29^^,^^30^^,^^40^^,^^41^^]^. India is a country with one of the highest number of low birth weight deliveries in the world^[^^42^^]^. Also, the country is witnessing increasing incidence of hypertension, diabetes over the last decade^[^^43^^]^. Therefore, it is logical to study renal volumes of newborns in such a country. Though sonographic studies for renal dimensions have been carried out in India, most of them are in children of different ages and the focus was not on kidney growth in SGA infants^[^^44^^-^^46^^]^. Though the study by Gupta et al. assessing renal dimensions in neonates, is informative, it included only AGA newborns^[^^47^^]^. 

 The limitation of our study is that some of the other confounding factors like maternal age, pre-conceptional weight and nutritional status could not be adjusted for, in the multivariable model, as these parameters were not available.

## Conclusion

This study brings forth the difference in kidney volumes between AGA and SGA newborns. Considering the future implications of being SGA and having low kidney volumes at birth, it is essential to have objective depiction of the relationship between these two vital parameters. Such a study of SGA newborns from the Indian subcontinent is expected to contribute substantially to the entire gamut of medical research concerning the role of intrauterine kidney growth in programming adult diseases.
